# Reviews of Books

**Published:** 1923-10

**Authors:** 


### REVIEWS OF BOOKS.

### FACTS AND THEORIES ABOUT CANCER.

**Cancer : Fallacy, Theory and Fact.** By John Shaw, M.D.Lond. (Routledge.)

Upon what is fallacy in relation to cancer it is given to no man to dogmatise. Every speculator and experimenter in this vitally important subject ought to wear the cloak of humility, for there is hardly a department of curative science in which, relatively speaking, so little has been definitely ascertained or in which so much is tentative. Dr. Shaw has failed to obtain the goodwill of most inquirers in this field, yet, speaking abstractedly, his theories are just as worthy of respectful treatment as those of any other man who has groped in the darkness to find the cause of cancer and its right treatment. It does not lie in the mouth of any researcher to declare *ex cathedrâ* that another's conclusions must needs be wrong, and, short of that co-ordination which it is the aim of the new British Empire Campaign to bring about, we are unlikely to add materially to our knowledge. We shall certainly not discover the cause of cancer, or how to cure it, by one supposed expert saying " You're another " to a second supposed expert, or to all of them in a body. However conscious we may be of the untenableness of each other's theories, and however completely convinced of the innate wickedness of those who disagree with us, we must be ready to realise that without many lines of inquiry, steadfastly followed even by those whose theories we suspect and whose methods we dislike, we shall be unlikely to reach the goal we all have in mind. It is not for us, therefore, to argue that Dr. Shaw's views are right or wrong. We do not know. But we do know that we ought to welcome any reasonably stated and fairly supported views on the subject even should they be shown in the end to be mere additions to the mountains of error.

Dr. Shaw set out to maintain two propositions—that operation is an irrational treatment for cancer and for tumours presumed to be in a pre-cancerous stage, and that operative zeal is responsible for the sacrifice annually of hundreds and thousands of men and women, and that cancer is curable without operation. After a wholesale criticism of modern methods of cancer-research and a recital of instances in which those responsible for such work have failed to see his side of the question, the author proceeds to review, from the standpoint we have indicated, cancer theories, vital statistics, cancer as a preventible and curable disease, &c. In short, the book proceeds in somewhat rambling fashion, with many references to personal disagreements, over a large part of the scientific fields which may affect the cancer problem.

We fear, however, that Dr. Shaw's way of expressing himself is not always such as to awaken sympathy. We are told, for instance, that the work of Dr. Murray and his colleagues of the Imperial Cancer Research is practically valueless—that statement is indeed tempered by the admission that Dr. Robert Bell and the author " utilised " the observation that a very moderate heat destroyed the vitality of cells of mouse tumours, so that the labours of that institution have not been entirely in vain. Both surgeons and the microscope come in for a measure of condemnation. As to the latter, Dr. Shaw asks, " What possible value can the microscope have in furnishing scientifically conclusive evidence of the cancerous nature of any tumour ? " This is backed up by the quotation of some utterances of great men. But let the reader consider that in Sir Samuel Wilks's pathological days the use of the microscope in investigating tumours, as we now employ it, did not exist. The essence of the modern method is fixation of tissues, cutting of thin sections, and differential staining ; these three things make all the difference. The microscope made by Ross in 1851 is quite capable of showing all that the pathologist desires to see in order to diagnose a tumour ; it is the arts of preparation, now well understood, that make the difference between an indecisive mess and a slide which gives a sure diagnosis.

Most readers will resent the suggestion, frequently repeated in this book, that surgeons operate primarily for love of filthy lucre. Here and there we find statements which will be generally accepted as true, though modifying some traditional dogmas, but they are hardly new to the modern medical student.

### MORE ABOUT SEX.

**Contraception : Its Theory, History and Practice.** By Marie Carmichael Stopes, D.Sc., D.Phil. (John Bale, Sons and Daniellson. 12s. 6d. net.)

**Sex Problems and Their Solution.** By Haydn Brown, L.R.C.P. (Mills & Boon ; 5s. net.)

**The Hygiene of Marriage.** By Isabel Emslie Hulton, M.D. (Heinemann ; 3s. 6d. net.)

We do not derogate from the importance of the subject when we say that we are in some danger of having too many books about sex and its problems, and the right conduct of marriage both physiologically and psychically. That there was need of simple common sense instruction upon these subjects is unquestionable, and the greatness of the need is, perhaps, indicated by the very large sale of Dr. Stopes's books. What was almost a " conspiracy of silence " lasted far too long. Little warning handbooks about the dangers of the budding sex-life there were in plenty. Their intention was excellent, but their note was namby-pamby rather than virile ; enough was said to excite curiosity, but not enough to allay it. Young people, whether about to be married or not, and young couples already married, were in real need of instruction upon what we may bluntly describe as the mechanics of sex, and the need has now been very fully supplied. Knowledge of this kind does not come by intuition, and a right and reverent understanding can but elevate the ideals of marriage and save a great deal of misunderstanding, repulsion, and actual misery. But it is almost time to call a halt—there is, at present, little room for more " popular " literature on the subject.

The three volumes before us are not, properly speaking, " medical books." They are addressed, not *ad clerum*, but to ordinary sensible men and women who understand the importance to themselves and the world of a sane view of the primary function of mankind, and how much there is at stake in redeeming it from the often ignoble associations which have been thrust upon it by that strange human perversity which takes pleasure in debasing the beautiful. Yet they are all backed by medical authority. Thus Dr. Stopes's book, in addition to a preface by Sir William Bayliss, has introductory notes by Sir James Barr, M.D., Dr. C. Rolleston, and Dr. Jane Hawthorne. Sir William Bayliss points out that there is in no language any similar book giving a complete history of methods of birth control, with full documentary evidence and a scientific account and criticism of the various systems of contraception. The author calls it "a manual for the medical and legal professions," and of its scientific value there can be no question. It is a large, learned, and laborious piece of work which brings together in comprehensive form nearly everything of importance that has been written or argued upon a difficult and delicate subject upon which opinions are widely divided. It has often been objected that the publication of detailed practical information upon methods of birth-control must necessarily lead to the increase of sexual immorality. But most knowledge is capable of abuse, and, after all, the vast majority of people desire to lead, and do lead, decent lives and are not deflected from the path of virtue by knowledge of how to sin with safety. This is an obscurantist type of argument for which there is no longer any room. For objections to control based upon religious conviction we must needs have the utmost respect, but those objections have much too often been mixed up with crude notions of physiology which have deprived them of much of their effect. Nature will neither be robbed nor flouted, and there are very many cases in which control, exercised without either robbery or flouting, is a duty alike to the parties and to the community. Dr. Stopes discusses this important question sanely and scientifically; and, indeed, this highly important question cannot be studied completely and dispassionately without reference to her distinctly remarkable book.

When we come to Mr. Haydn Brown we are in a different atmosphere. He is a "breezy writer," with plenty of what is called "horse-sense," yet with a clear conviction that if you do not agree with him you are in a very bad way. "The reader," he says squarely, "must accept on broad biological and ethical grounds what is written in these chapters as belonging to at least average sense. If any disagree then they must be either abnormal in their reckonings, or must have particular reasons for being exceptional." Thus threatened with being regarded either as a monstrosity or a bad lot, most of us will hasten to come in out of the way of Jove's thunderbolts. But indeed there is little need to take cover. Mr. Haydn Brown digresses a good deal and he is sometimes in the air, but nearly everything that he says about sex and modesty, sex perversion, love, and such-like subjects we may readily accept. Our difficulty, which we fear is fundamental, is that we fail to see any particular reason for saying it.

Dr. Emslie Hutton's little book is, from first to last, excellent; and we share the admiration of Dr. A. Louise Milroy, who writes the Preface, for "the way in which she has so delicately expressed the necessary information." Her object, indeed, has been almost entirely informative, and she tells the things that ought to be known simply and naturally in the way that a sympathetic and understanding medical man might put them in conversation. Nothing is omitted and nothing unduly dwelt upon. Our only criticism is that Dr. Hutton's remarks on contraceptives will seem very elementary to any reader fresh from the perusal of Dr. Stopes's detailed and technical book.

### A NEW TUBERCULOSIS TREATMENT.

**A Simple Treatment for Tuberculosis.** By Owen F. Paget, M.D. With an introduction by J. George Adami, M.D., F.R.S., and Prefatory Remarks by W.P. Birmingham, B.A., M.D. (Constable: 5s. net.)

Still they come, these new treatments for tuberculosis, and there are few things so old as new treatments for this disease. "Spes phthisica" may well be defined as a condition occasionally observed in consumptives and invariably found in an acute form in the advocates of new cures for consumptives. The author is well advised in his choice of title; the treatment seems to be perfectly simple, and consists of insufflating dried, powdered, tubercle bacillary emulsion on to the epithelial cells of the ethmoid, superior turbinates and elsewhere in the nose. The tuberculin used is the B.E. prepared by Parke, Davis & Co., and the doses employed range from 1/6,000 mgm. to 1/40 mgm. of the bacillary emulsion.

The statistics given in support of this treatment are referred to in the introduction of this book supplied by Prof. J. G. Adami, who writes that ". . . while among the Australian soldiers in the tuberculosis wards there were three deaths in the fortnight before this treatment was undertaken, during the ensuing ten months, with treatment, there were only two deaths among the 500 and more admitted to those wards, on the diagnosis of other medical officers, as suffering from tuberculosis or suspected tuberculosis." Statistical evidence of this kind is reminiscent of the story of a laryngologist who told a patient suffering from tuberculosis of the larynx that he would assuredly recover. Asked for his reasons for giving such a happy prognosis, the laryngologist replied "Nineteen out of every twenty patients die from this disease, and you are my twentieth patient." Every conscientious critic of new methods of treating tuberculosis is haunted with the fear that one day he may damn a treatment which is really a cure, and that instead of treating angels unawares he may have slammed the door on them.

Dr. Paget's treatment may, indeed, be the cure for which millions have waited in almost breathless suspense, and this is not the time or the place to pass judgment on it. But it is both justifiable and desirable to point out at once that the evidence for his case, as given in Dr. Paget's book, is unconvincing. The author shows no profound knowledge of tuberculosis and its literature. Ignorance of much of the literature is, perhaps, a virtue, for most of it is nebulous and misleading stuff. Inadequate knowledge of the disease itself is a more serious matter. The author is to be congratulated on his clear account of the technique which requires little skill. It is to be hoped that his treatment will be given a fair trial in this country, and that if and when experts find it of value after full and prolonged trial, they will report on it in such a way that their conclusions will bear close scrutiny.

### AUTO-SUGGESTION AND MENTAL HEALING.

**My Method : Including American Impressions.** By Emile Coué. (Heinemann. 5s. net.)

**Talks on Psycho-Therapy.** By William Brown, M.D., D.Sc., M.R.C.P. (University of London Press. 2s. 6d. net.)

**Hypnotism and Treatment by Suggestion.** By Albert E. Davis, F.R.C.S., L.R.C.P. (Putnam. 5s. net.)

M. Coué is a very modest man, and he was quite alarmed when the reporters who hurried on board the "Majestic" to interview him on the occasion of his recent visit to the United States addressed him as "Doctor" and "Professor." "I was obliged," he says, "to correct them with reminders that I am not a doctor; I am not a professor." Still more is he concerned to point out that he is not a healer, and that it is not in the least necessary to come into personal contact with him to obtain beneficial results :—

"I do not claim to have invented anything. I have merely reduced to a simple formula for everyday use and practice theories which were known to be truths thousands of years ago. Still less have I invented a new faith, as some would appear to infer. One day a gentleman, interviewed by one of the newspapers, described my method of auto-suggestion as a 'direct challenge to the Church.' I confess I fail to see any relation between religion and auto-suggestion. Is medicine a challenge to the Church? . . . Other religious leaders look askance at auto-suggestion because it has come to be associated with alleged 'miracles' which I am supposed to have worked. Now, miracles do not exist. I have never accomplished any and never shall. As a matter of fact, the so-called 'miraculous' cures are the simplest and the most easily explained of all. They prove that, actually, the sufferers only thought they were sick. Thoughts produced (or prolonged) the symptoms; and in that respect they were really sick. But directly they were made to realise that their ills could be overcome by imagination they were cured."

It is possible to disagree profoundly with M. Coué, but it is quite impossible to dismiss him lightly. In *Talks on Psycho-Therapy*, Dr. Brown gives us much interesting matter in very limited space. Delivered in the form of three lectures at King's College, London, the book, as he himself says, is "not of the popular variety nor even quite elementary." It would certainly be more or less incomprehensible to a complete stranger to the subject, but by the beginner as well as by the more advanced student of these matters this attempt to outline the present position in psycho-therapy should be heartily welcomed. Dr. Brown has the gift of clear exposition—his brief description of the theories of Freud and Juny, for instance, is a masterpiece of compression.

Dr. Davis's book has reached a fourth edition, and he has now revised and enlarged it. As Hon. Physician to the Liverpool Psycho-Therapeutic Clinic he has achieved many remarkable results in treating cases of shell-shock, and he writes so well and with such a pleasant absence of unnecessary technicalities that the popularity of his book is readily understood.

### THE MANCHESTER HOSPITALS.

**History of St. Mary's Hospitals, Manchester, 1790-1922.** By John Webster Bride, M.D. (Sherratt & Hughes, Manchester: 6s.)

This welcome addition to hospital histories deals not only with the story of the institutions, but includes biographies of some of the medical staff. Written by one of them, the present Registrar, it differs from most books of the kind in emphasising the medical aspects of the history, to which the administrative side is made subordinate, so that for once we escape from the familiar tale of debts and appeals and extensions, to concentrate on such dates as those in which the Cæsarian operation and ovariotomy were first performed, and to say a word or two about the surgeons responsible for them. This undeniably lends a freshness to the sketch which is very welcome.

St. Mary's was originally a lying-in hospital, though much was first made of the practice of attending the mothers in their own homes, no doubt to avoid the risk of their contracting puerperal fever. Till 1849 inoculation for cow-pox was regularly practised, and we learn how subscriptions suddenly increased in 1837 because there were three elections of surgeons in that year. The subscription gave the right to vote, and the candidates, no doubt at Manchester as elsewhere, had their several parties and backers. The hospital claims to have been the first to give systematic instruction to nurses, and as a centre of teaching was active from the first. Not till 1861 was a lay secretary appointed to relieve the house surgeon, hitherto responsible, of administrative work. In 1846 Charlotte Brontë took her father to be operated on for cataract by W. J. Wilson, F.R.C.S., who had held the post of man-midwife-in-ordinary to the hospital from 1815 to 1824.

The illustrations give a good idea of the different buildings occupied by the hospital at its respective stages of growth, and the portraits of the surgeons are of great interest. In fact, the method adopted by Dr. Bride gives to his small volume an interest, a life and a variety often missed by longer works which rely too much on old minute books and reports and omit the sort of character to be found in the personalities who compose a hospital's staff.

---

### MISCELLANEOUS NOTICES.

#### Hints on Massage.

Massage in Fractures and Injuries to Joints. By Olive F. Sands. (The Scientific Press. 1s. 3d. net.)—Intended as a help to trained masseuses who have not had the opportunity of recent experience in surgical treatments, and for students this is a useful addition to the Pocket Guide Series.

#### Parasitic Diseases.

Animal Parasites and Human Disease. By Asa C. Chandler, M.S., Ph.D., Instructor in Biology, Rice Institute, Houston, Texas. 2nd Ed. (Chapman and Hall. 22s. net.).—This is an example of the very useful type of semi-popular scientific work which finds ready sale in America. As the author says, the popular ignorance of important facts of parasitology and preventive medicine, even the facts which have been common bases of operation for scientists for many years, is deplorable. He certainly gives his readers opportunity to remedy their own ignorance, and his chapters are carefully and clearly set down. The facts quoted are nearly all likely, at some time, to be of direct importance to every one of us, even though we do not live in subtropical lands, and for the traveller, the farmer or merchant who is interested in the progress of science and civilisation, Dr. Chandler has provided a thoroughly useful book.

### For the Invalid.

A Silver Lining. By G. H. A. Rives. (W. J. Bryce. 2s. 6d. net.). This little book has been written by an invalid in the hope of cheering other invalids and of helping them to feel that life is still worth living. If a trifle exuberant, *A Silver Lining* is far more robust than most books intended for the sick and is lacking neither in originality nor imagination. It is clear that the author still finds life full of interest and emotion, and that in itself should be stimulating to readers of these little essays.

### A Text-book of Public Health.

Hygiene and Public Health. By Louis C. Parkes, M.D., D.P.H., and H. R. Kenwood, C.M.G., M.B., D.P.H. 7th Edition. H. K. Lewis & Co. 20s. net.—It is almost unnecessary to commend this invaluable text-book, which now passes into its seventh edition, to Students taking the D.P.H. Course, for whom it is essential. One change of some importance is noted. The chapter on Sanitary Law and Administration is now omitted, as the subject is too wide for brief treatment, and Public Health Students are advised by the authors to consult some book entirely devoted to this subject. In the place of this section there is introduced into the volume much new and interesting matter. The authors are to be congratulated on the way in which, while giving a mass of information, scientific and otherwise, they avoid language which is too technical. The book reads easily, is well indexed and illustrated, and ought to find a place on the book shelves of members of Public Health Committees and of all others similarly interested; and it will be repeatedly taken down for reference.

### A Compendium of Nursing.

Pocket Cyclopedia of Nursing. Edited by R. J. E. Scott, M.A., B.C.L., M.D. New York. (The Macmillan Company, 1923. 14s. net.).—The contents of this book, which is edited by a doctor, are, as stated in the preface, almost entirely composed of "material written by nurses for nurses," in some cases specially contributed, in others taken from books by American medical or nurse authors. While appreciating to the full the great difficulties of making suitable selections for such a work, the ordinary reader on looking through its pages will feel a trifle perplexed as to whether it is intended for the trained nurse or for the probationer. The lengthy instructions given for bed-making, bandaging and the treatment of bed sores seem to point to a text-book for the latter; but if it is meant for trained nurses, we venture to think they will be disappointed to find no mention of insulin in the article on diabetes, of Dreyer's treatment for tuberculosis, or of the vitamines in an anti-rachitic diet. With this exception the book is useful as a handy work of reference, is well tabulated and of convenient pocket size.

### Salisbury Infirmary.

The History of Salisbury Infirmary. By Charles Haskins. (The Infirmary League. 5s.).—In 1893 appeared the "Records of the Salisbury Infirmary, 1766-1893," by the Rev. T. J. Woodall, the then chaplain, which Mr. Haskins, already known as a local historian, has used in the compilation of the present book. This first appeared as a series of articles, which deserved reprinting for the interest that the extracts from the old minute books give to the story. On these stores of humour and information Mr. Haskins has freely drawn and has therefore provided a more detailed and vivid picture than can always be relied on in such histories. The book is enriched with fourteen portraits and other illustrations, some after Hoppner and Gainsborough, and cannot fail to interest either local supporters or the wider circle to whom institutional history appeals. Apart from its local work, Salisbury Infirmary has its place in history in regard to such men as Jenner and Mr. Roosevelt, who wrote a letter of thanks from the White House for its services to the Americans who suffered in the railway accident that occurred in 1906. The treatment is detailed and thorough and makes a welcome addition to hospital literature.

### The Care of Infants.

La Thérapeutique du Nourrisson en Clientèle. Par Professeur P. Nobécourt et Dr. M. Maillet. Paris: A. Maloine et Fils.—This compact volume of 864 pages contains a very comprehensive review of all the ills to which flesh is heir at the beginning of life. Useful hints are given as to the various ways in which drugs can be administered, and the general principles guiding the physician in the conduct of the case are clearly outlined. A comparison of this work with text-books on children's diseases of only a decade or two ago cannot fail to impress the reader with the advances that have been made in this field. The problem of diphtheria, for example, is well on the way to a satisfactory solution, thanks to the Schick reaction, and this is only one instance of many showing how the expectation of life in early infancy has become much better than it was less than a generation ago.

### Abdominal Troubles.

The Early Diagnosis of the Acute Abdomen. By Zachary Cape, M.D., M.S., Surgeon to Out-Patients, St. Mary's Hospital, etc. Illustrated. 2nd edition. (Henry Froude and Hodder & Stoughton; 12s. 6d. net.). Les Désanglés du Ventre. Par le Docteur L. Chauvois, Ancien Interne, Lauréat de la Faculté et des Hôpitaux de Paris. Illustrated. (Maloine, Paris; 8 francs.)—Only two years have elapsed since the first publication of Dr. Cape's book. Few alterations have been made in this edition, but the section on superficial hyperæsthesia has been rewritten and brought up to date in the light of more recent investigations. It still, however, remains an uncertain guide to abdominal diagnosis. The information is accurate and concise, the illustrations are simple, clear and diagrammatic. The book should serve as a valuable guide to both student and practitioner in the diagnosis of acute abdominal disease in its early phases. Dr. Chauvois deals with ptosis of the abdominal organs, and his book is intended for the practising physician as well as the public in general. Dr. Chauvois regards it as a widespread deformity originating in early life and emphasises the importance of early and preventive treatment. This book is abundantly illustrated and forms interesting and attractive reading.

### The Microscope in Disease.

Practical Morbid Histology. By Robert Donaldson, M.A., M.D. (Heinemann. 15s. net.).—As Sir Humphry Rolleston, K.C.B., the President of the Royal College of Physicians, remarks, in a preface, this book is not one for the armchair. It is, rather, a handbook for the earnest student of the morbid structural changes met with in disease, and as such it is intended by the author to be used while actually working with the microscope and in the post-mortem room. In sixteen chapters the morbid appearances of the various organs and tissues are described: first with regard to the changes visible to the naked eye, and next as to their histological manifestations. This arrangement is a satisfactory one from the student's standpoint, and it will also be found useful to the busy practitioner who needs to refresh his memory prior to the performance of an autopsy. The various epithelial and connective-tissue tumours are described in separate chapters, as are also the melanomas, endothelial tumours and those of mixed origin. The division of the carcinomata adopted is that into three main groups, according to the predominant type of cell present—squamous, columnar and spheroidal celled. The author regards the presence of cysts in a breast which is already affected with cancer as being probably more than a mere coincidence and as a "pre-cancerous condition." In the examination of a section from a case of Paget's disease of the nipple, the student is directed to search for evidence of scirrhous carcinoma. Not the least valuable portion of the book is the chapter dealing with modern histological methods. In this are given the formulæ for the preparation of the principal stains, together with directions for section-cutting and for the preservation of museum specimens. In another appendix are set out the characteristics of the various worm-parasites and their ova. The book will be welcomed by teachers and students alike, and will prove a trustworthy guide to the intricate paths of morbid histology.

---

Sir Philip Sassoon, M.P., Hon. Treasurer of the Royal Northern Hospital, has received a donation of £400 from a gentleman who wishes to remain anonymous.

